# Tissue Resident and Migratory Group 2 Innate Lymphoid Cells

**DOI:** 10.3389/fimmu.2022.877005

**Published:** 2022-04-29

**Authors:** Laura Mathä, Fumio Takei, Itziar Martinez-Gonzalez

**Affiliations:** ^1^ Department of Microbiology, Tumor and Cell Biology, Karolinska Institutet, Solna, Sweden; ^2^ Terry Fox Laboratory, British Columbia Cancer, Vancouver, BC, Canada; ^3^ Department of Pathology and Laboratory Medicine, University of British Columbia, Vancouver, BC, Canada

**Keywords:** group 2 innate lymphoid cells, type 2 inflammation, tissue residency, recruitment, migration

## Abstract

Group 2 innate lymphoid cells (ILC2s) are present in both mouse and human mucosal and non-mucosal tissues and implicated in initiating type 2 inflammation. ILC2s are considered to be tissue resident cells that develop in the perinatal period and persist throughout life with minimal turning over in adulthood. However, recent studies in animal models have shown their ability to circulate between different organs during inflammation and their potential functions in the destined organs, suggesting their roles in mediating multiple type 2 diseases. Here, we review recent findings on ILC2 migration, including migration within, into and out of tissues during inflammation.

## Introduction – Tissue Residency of ILC2s

ILC2s reside at barrier surfaces, such as the skin, lung and intestine and are activated by cytokines released upon tissue damage, such as IL-33, IL-25 and thymic stromal lymphopoietin (TSLP) (1). They potently produce type 2 cytokines, IL-5 and IL-13, and initiate a cascade of reactions leading to type 2 inflammation. Owing to their ability to produce copious amounts of type 2 cytokines, they have been implicated in various type 2 inflammatory diseases, such as asthma and atopic dermatitis (2). The tissue resident nature of ILC2s was initially reported using parabiosis mouse models, where congenic mice were surgically joined together to investigate migratory capacity of cells. Gasteiger et al. demonstrated that more than 95% of ILCs in small intestine (SI), salivary gland, lung and liver are tissue resident with minimal trafficking at homeostasis (3). Interestingly, there was a slight increase in ILC2s derived from the paired parabiont in a long-term parabiosis experiment (3), suggesting some degree of ILC2 replenishment from hematogenous sources, but to a much lesser extent compared to circulatory lymphocytes, such as NK, T and B cells. They also demonstrated tissue residency of ILC2s in inflammatory conditions by using a mouse model of *Nippostrongylus brasiliensis* (Nb) infection in parabiotic mice. During acute Nb infection, ILC2s locally expanded and very little influx of ILC2s derived from the other parabiont was observed. In contrast, minor but significant recruitment of ILC2s was observed during the chronic infections. Moro et al. also reported local expansion of tissue resident ILC2s in fat-associated lymphoid clusters (FALC), lung and bronchoalveolar lavage fluid (BALF) during respiratory inflammation induced by intratracheal IL-33 administration into parabiotic mice (4). Many other groups also used parabiosis models and confirmed ILC2 tissue residency at homeostasis and during inflammation (5–8).

## Tissue Seeding and Adaptation to the Tissue Environment

**Figure 1:**
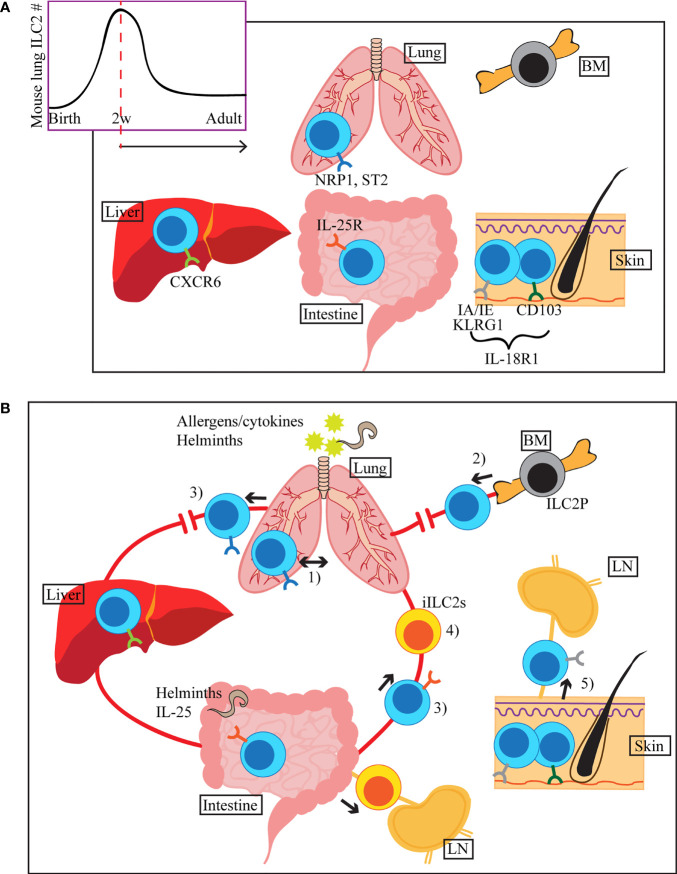
**(A)** ILC2s seed tissues early during the neonatal period, with a peak of ILC2 accumulation around 2 weeks after birth (mouse lung). Once in tissues, they receive environmental cues and adapt tissue specific phenotypes, indicated by different colours of the surface receptors in the figure. Common ILC2 markers in each tissue are shown, but they can also be expressed in other organs. **(B)** ILC2s migrate within the lung upon i.n. IL-33 or Aa treatment (1). During allergen-induced respiratory inflammation or tissue disruption, ILC2s are recruited from hematogenous sources (2). When activated by allergens/cytokines or helminth infection, some of the lung and small intestinal ILC2s appear in the peripheral blood (3). Stimulation by i.n. IL-33 or papain induces migration of a subset of lung ILC2s to the liver, where they contribute to the regulation of local immunity (3). Upon helminth infection or i.p. IL-25 stimulation, small intestine-derived inflammatory ILC2s (iILC2s) appear in circulation and accumulate in the lung, liver, mesenteric LN and spleen (4). iILC2s ultimately become conventional ILC2s in the lung. Tissue resident and circulating ILC2s are present in the skin. During atopic dermatitis-like inflammation, circulatory ILC2s migrate to the draining LN (5).

ILC precursors and ILC2s can be detected in the fetal intestine, lung and skin during embryonic development in mice (7, 9), while the majority of adult ILC2s seems to be neonatally derived (**Figure 1A**). Several studies showed that ILC2s are rare in mouse lungs right after birth, but they gradually increase in number, reaching a peak around two weeks after birth (10–12). Knock out mouse studies and antibody blocking showed that this ILC2 expansion is dependent on IL-33 and IL-7 signaling (7, 11, 12). The defect in ILC2 expansion in the absence of IL-33 signals is likely due to the impairment of ILC2 progenitor (ILC2P) egress from the bone marrow (BM), as ST2 deficient mice have increased numbers of ILC2P in the BM in parallel with reduced number of ILC2s in the lung 2 weeks after birth (6). The neonatal wave of ILC2 proliferation and activation is not only limited to the lung but seems systemic as high percentages of Ki67^+^ and IL-5^+^ ILC2s were observed in the SI and skin as well (7). Schneider et al. used tamoxifen-inducible Cre approach to irreversibly label prenatal and postnatal ILC2s and showed that the majority of adult lung, visceral adipose tissue and ST2^+^ SI ILC2s develop in the neonatal period while a small fraction of prenatally-derived ILC2s contributes to the adult pool of ILC2s (7). In contrast, the proportion of the neonatally-derived skin, BM and IL-25R^+^ SI ILC2s rapidly declined, suggesting that the turnover rate of ILC2s differs depending on the tissue environment (7). Overall, this and our own studies detected very few ILC2s slowly turning over in adult mice (7, 13, 14).

Once in peripheral tissues, ILC2s are phenotypically and functionally adapted to the tissue specific environment of the residing organs. This was shown by single cell RNA sequencing (scRNAseq) analyses of ILC2s isolated from various adult mouse organs, which revealed tissue-specific transcriptomic signature of ILC2s (15). For instance, intestinal ILC2s have enhanced expression of *Il17rb* and *Ikzf3* (encodes Aiolos) and lung ILC2s express *Nrp1*, while skin ILC2s highly express *Il18r1* (15, 16). Interestingly, ILC2 turnover is strikingly different depending on their residing tissues, further supporting tissue specific dynamics of these cells. The ILC2 pool is relatively stable in the lung, SI and adipose tissues, whereas ILC2s are quickly replenished by newly generated pool of ILC2s in the skin and BM (7). It is possible that each tissue has its specific ILC precursor populations that contribute to the maintenance of ILC2 pool at homeostasis and ILC2-poiesis during inflammation. Indeed, recent work has identified the presence of IL-18R1^+^ ST2^-^ tissue ILC precursors in mouse lung (17, 18).

It is important to note that tissue distribution of ILC2s differ depending on the species. In the human system, helper ILCs, including ILC2s, are underrepresented in the non-mucosal compared to the mucosal tissues due to the abundance of NK cells, similar to mice (19). However, ILC2s are relatively rare in human SI (20), whereas they are one of the major ILC populations in mouse SI (8, 21).

## ILC2 Recruitment During Inflammation and Tissue Disruption

Although Gasteiger et al. demonstrated ILC2 tissue residency under steady-state and acute inflammatory conditions using parabiotic mice, they did observe significant infiltration of donor-derived ILC2s during chronic Nb infections (3). These data suggested potential recruitment of ILC2s from hematogenous sources in chronic inflammatory conditions. Karta et al. showed that ILC2 numbers were reduced in the BM after intranasal (i.n.) treatment of mice with the fungal allergen, *Alternaria alternata* (Aa). In parallel, they observed ILC2 increase in the blood and lung, suggesting that ILC2s emigrate from the BM, circulate and are recruited to the lung during airway inflammation (22). This recruitment was mediated by β2 integrin. Stier et al. similarly used Aa induced lung inflammation model to show elevated IL-33 levels in the serum, and IL-33 dependent egress of ILC2P from the BM (6). Moreover, systemic treatment of mice with intravenous IL-33 injections caused egress of ILC2P from the BM partially through downregulation of CXCR4 (6). These data suggest that, in Aa induced inflammation, ILC2P may be mobilized in response to IL-33 release by regulating β2 integrin and CXCR4 expressions.

In the same study, Stier and colleagues used the parabiotic system, where one parabiont was irradiated before parabiosis surgery, to model tissue disruption (6). ILC2s derived from non-irradiated parabiont readily reconstituted the irradiated parabiont, illustrating the ability of ILC2s to migrate and fill the empty niche. A similar observation has been made in the human system, where ILC2 reconstitution occurred in severe combined immunodeficiency (SCID) patients who underwent hematopoietic stem cell transplantation (HSCT) in myeloablative conditions (23). This suggests that ILC2 recruitment to an empty niche may occur upon tissue disruption, which likely provides signals for ILC2-poiesis and recruitment. However, it is unclear whether ILC2s can repopulate the empty niches under homeostatic condition. When parabiotic mice were generated between ILC2 sufficient and deficient (*Il7r* ^-/-^, also deficient in T and B cells) mice, ILC2s did not efficiently reconstitute the deficient host (7).

The idea of ILC recruitment is supported by some human studies. CD117^+^ ILCs in human peripheral blood have been described as circulating ILC precursors that can give rise to all ILC subsets *in vitro* and *in vivo* upon transfer into immunodeficient mice (24). Authors suggested that these ILC precursors can generate tissue ILCs when required in steady-state or during inflammation. In another study, scRNAseq of blood and tissue ILC2s demonstrated that blood ILC2s express cell trafficking associated genes, such as *S1PR2* and *CCR2*, whereas lung ILC2s have activation-related phenotype characterized by the expression of the receptors for ILC2 activating cytokines, *IL1RL1* and *IL17RB* (25). This observation led authors to hypothesize that blood ILC2s may be recruited to the lung, where they become responsive to epithelium-derived cytokines. Stimulation of blood ILC2s with ILC2-activating cytokines indeed induced IL-1RL1 (IL-33 receptor) and IL-17RB (IL-25 receptor) expression in blood ILC2s, which resemble the phenotype of lung ILC2s. It has also been shown, in asthmatic patients, that ILC2s accumulate in BALF after allergen challenge, whereas circulating ILC2 numbers decrease at the same time, suggesting that ILC2s are recruited from circulation into the lung during inflammation (26).

Overall, despite their tissue resident nature, existing data indicate that ILC2s can be *de novo* generated and recruited to tissues to meet the production demand, during inflammation and tissue disruption. The disparity between the reports demonstrating local expansion of ILC2s and those that show ILC2 recruitment during inflammation can be on account of several factors. First of all, the majority of the studies that demonstrated tissue residency of ILC2s were in mouse models of Nb infection or IL-33 treatment in parabiosis mice (3–5, 8), whereas those that showed recruitment were Aa model in non-parabiotic mice, where ILC2 recruitment was determined based on decrease in ILC2P/ILC2 in the BM (6, 22). Stier et al. saw an increase in IL-33 in the serum after Aa treatment (6), while we did not detect it after i.n. IL-33 treatment (8), indicating that the inflammatory events that occur upon the initiation of immune responses can greatly differ depending on the model. The strain of mice and identification of ILC2s are also inconsistent among these papers, and consequently, we cannot directly compare different studies. Lastly, the time point of the analyses is determined by the models and thus differs in each study. Therefore, considering all of the potential variables among these reports, it is likely that recruitment and local expansion of ILC2s are not mutually exclusive events during inflammation but rather they may occur sequentially or concurrently. It is also possible that in some cases, local expansion of ILC2s dominate, while recruited ILC2s are the primary responders in other cases.

## ILC2 Migration During Inflammation

As the first line of defense in barrier tissues, ILC2s exhibit dynamic behaviour within tissues upon exposure to damage signals. Recent evidence also suggests that a subset of ILC2s may leave their residing tissues, circulate and migrate to a different organ(s) once activated (**Figure 1B**).

### ILC2 Migration Within Organs

ILC2s in multiple organs, including the lung, liver, brain meninges and adipose tissue, reside in perivascular adventitial cuff spaces (27, 28). In the lung, the majority of the ILC2s are in proximity to adventitial stromal cells, which produce IL-33 and TSLP (27). However, i.n. IL-33 or Aa treatment in mice induces ILC2 motility around blood vessels and airways, where ILC2s exhibit amoeboid-like movement (29). Interestingly, ILC2s present much more motile behaviour compared to CD4^+^ T cells in these models. Intra-organ motility and activation of lung ILC2s are dependent on chemotactic signal through CCR8 in IL-33 treated mice, whereas its ligand CCL8 also induces migration of human ILC2s. TGF-β has also previously been shown to increase the basal motility of ILC2s in mice (30). The induction of ILC2 motility during IL-33 mediated inflammation was, at least in part, due to collagen I in the extracellular matrix (ECM), which provides signals to alter ILC2 shape towards pro-migratory phenotype with polarized F-actin arrangement (29).

Dahlgren et al. previously reported that ILC2s primarily remain in the adventitial cuff spaces during inflammation induced by Nb infection, papain treatment or systemic IL-33 injections, while a small proportion of them also expands in the parenchyma (27). In the recent report by Cautivo et al., the authors closely examined the topographic distributions of ILC2s during mixed inflammation induced by exogenous IL-33 + IFNγ treatment or Nb + *Listeria monocytogenes* infections (28). Although the majority of ILC2s reside near the adventitial space in steady state, type 2 inflammation induced their expansion in the parenchyma. In the mixed inflammation models, IFNγ restricted parenchymal ILC2 expansion and promoted their cell death. RNAseq analyses revealed that IFNγ suppressed cell survival and movement associated pathways. Moreover, IL-33 repressed ILC2 retention-associated genes, such as *Cd44* and *Cd69*, while it induced cell trafficking-related genes, including *S1pr1*, *S1pr4*, chemokine receptors and integrins. The authors suggest two potential mechanisms by which ILC2s traffic to the parenchyma. They either migrate from the adventitia to parenchyma, or they enter directly from circulation into parenchymal areas, which requires further investigation. However, lung and liver ILC2 accumulation in the parenchyma was inhibited by blockade of S1PRs, suggesting that the latter may be the dominant mechanism.

### ILC2 Emigration to Other Organs Upon Activation

Although ILC2s are rare in naïve mouse blood, they are readily detectable in healthy human peripheral blood (19, 20). Circulating ILC2s in healthy human blood are considered naïve as they express the CD45RA isoform of the CD45 receptor (31), which is expressed by naïve T cells (32). Previous studies reported that the frequency of ILC2s is elevated in the peripheral blood of patients with allergic diseases, such as asthma (33). Therefore, it is possible that these ILC2s are in circulation en route to the site of inflammation as described earlier. However, it is also likely that these ILC2s have left their residing tissues after being activated. Indeed, several recent mouse studies have described activation induced migratory behaviour of ILC2s. Intraperitoneal (i.p.) injections of mice with IL-25 induces an expansion of KLRG1^+^ST2^-^ ILC2s, also known as inflammatory ILC2s (iILC2s), in the lung, liver, mesenteric LN and spleen (34). These cells are rare in naïve mice but are elicited by IL-25 stimulation and Nb infection. They are transient cells appearing at an early stage of inflammation and eventually become ST2^+^ conventional ILC2s in tissues. In a later study by the same group, it was found that iILC2s are circulatory cells that originate in the intestine and migrate in a sphingosine 1 phosphate (S1P) dependent manner upon IL-25 stimulation or Nb infection (5). It is important to note that a human equivalent of mouse iILC2s has recently been identified in the nasal polyps (NP) of the patients with chronic rhinosinusitis with nasal polyps (CRSwNP) (31). These ILC2s exhibited activated gene expression profiles and were marked by the expression of CD45RO, which is a well-established marker of activated T cells (32). CD45RO^+^ ILC2 were enriched in the gene signature of mouse iILC2s (31). Interestingly, these CD45RO^+^ ILC2s were not only present in the inflamed tissues, but also increased in the peripheral blood of the CRSwNP patients compared to healthy controls, suggesting that they may have migrated out of the inflamed nasal tissue.

Ricardo-Gonzalez et al. investigated ILC2s that appear in circulation upon Nb infections in mice (35). Circulatory ILC2s elicited after Nb infections exhibited two distinct phenotypes at different time points during the course of infection. The first circulatory ILC2 wave, which appears on day 5 after infection, was accounted for by IL-25 dependent SI ILC2s. In contrast, the second wave on day 12 was due to IL-33 dependent lung ILC2s. Migration of ILC2s was S1P dependent. These results indicated the potential of ILC2s residing in various organs to invade circulation upon activation and propagate local immune response to systemic type 2 immunity.

Nakatani-Kusakabe et al. recently performed scRNAseq of skin and draining LN of IL-33 transgenic (IL33tg) mice (36). These mice express IL-33 under the control of keratin 14 promoter and spontaneously develop atopic dermatitis (AD)-like skin inflammation (37). They identified two clusters, cluster 1 and 2, of ILC2s in the skin, while only cluster 1 was present in the draining LN. They hypothesized that only cluster 1 is migratory, and thus named the two clusters “circulating” and “resident” ILC2s, respectively (36). To track ILC2s, they crossed IL33tg mice with photoconvertible KikGR knock in mice, which upon exposure to violet light, undergo irreversible photoconversion from green to red fluorescence (38). Using these mice, they labeled skin cells and analyzed skin-derived and LN ILC2s in the LN by scRNAseq (36). Interestingly, skin (red) and LN (green) ILC2s shared gene signatures, suggesting that LN ILC2s are a single subset of migratory ILC2s that have migrated from the skin, while skin ILC2s consists of resident and migratory ILC2s. The “circulating” ILC2s uniquely expressed IA/IE and KLRG1, while only skin “resident” ILC2s expressed CD103, which is a known marker of skin ILC2s (39). While both subsets can produce type 2 cytokines, skin “resident” ILC2s have a higher capacity to produce them compared to the “circulating” subset.

We recently followed lung ILC2s after activation by i.n. administration of IL-33 or the protease allergen papain to determine the destination of migratory ILC2s (8). Similar to the studies described above, ILC2s quickly appeared in circulation after activation in the lung and their migration was dependent on S1P signaling. ILC2 numbers also increased in the spleen and liver, but not in the SI. Interestingly, liver ILC2 numbers remained higher than those in naïve mice even a month after initial activation in the lung. Lung-derived ILC2s promoted fibrotic inflammation in the liver in an IL-33 induced type 2 inflammation model, whereas they exerted protective effects in concanavalin A induced acute hepatitis model (8). Based on the phenotypic analyses, we determined that the migratory ILC2s are a subset of ILC2s which expresses the chemokine receptor CXCR6, a surface molecule readily detected on the liver resident ILC2s. In contrast, they lack the expression of CD103, which is highly expressed by the activated lung ILC2s (8). ILC2s that accumulated in the liver after i.n. IL-33 treatment also produced more IL-6 compared to lung ILC2s. This study highlighted the possibility of migratory ILC2s to mediate inflammatory events in two different organs and potential connections between various allergic diseases. However, the physiological contribution of the migratory ILC2s in linking several human diseases is currently unclear and requires further investigation.

## Conclusion and Future Perspective

ILC2s are widely accepted as tissue resident lymphocytes under steady-state and inflammatory conditions. Hence, the majority of the studies in the field has focused on the pathological or protective roles of ILC2s in the sites where they reside. However, recent evidence in mouse models highlight the capacity of ILC2s to circulate and migrate between tissues upon activation, which is supported by evidence from human studies. This opens up a whole new avenue of research investigating connections between various type 2 immune mediated diseases, which is currently lacking. Understanding the mechanisms by which ILC2s traffic between tissues, heterogeneity among tissue resident and migratory ILC2s and their functions will be key areas of investigation in the future research, which may lead to the development of therapies specifically targeting the migratory populations of ILC2s.

## Author Contributions

LM wrote and edited the manuscript and generated the figure. FT and IM-G reviewed the drafts, provided critical input, and edited the manuscript and figure. All authors contributed to the article and approved the submitted version.

## Funding

This work was supported by grants from the Canadian Institutes of Health Research (GR018902) and the Knut and Alice Wallenberg Foundation (2019.0221).

## Conflict of Interest

The authors declare that the research was conducted in the absence of any commercial or financial relationships that could be construed as a potential conflict of interest.

## Publisher’s Note

All claims expressed in this article are solely those of the authors and do not necessarily represent those of their affiliated organizations, or those of the publisher, the editors and the reviewers. Any product that may be evaluated in this article, or claim that may be made by its manufacturer, is not guaranteed or endorsed by the publisher.
